# Hybrid attention-based multi-class classification of Ethiopian legal texts using deep learning

**DOI:** 10.1371/journal.pone.0348805

**Published:** 2026-05-13

**Authors:** Amlakie Aschale Alemu, Malefia Demilie Melese, Daniel Arega Mengesha, Misganaw Aguate Widneh

**Affiliations:** 1 Electrical and Computer Engineering, Gafat Institute of Technology, Debre Tabor University, Debre Tabor, Ethiopia; 2 Department of Information Technology, Gafat Institute of Technology, Debre Tabor University, Debre Tabor, Ethiopia; Wuhan Institute of Technology, CHINA

## Abstract

The exponential growth of legal documents in Ethiopia has created an urgent need for efficient and accurate automated classification systems tailored to the country’s unique linguistic and legal contexts. This study presents an enhanced deep learning approach for multi-class classification of Ethiopian legal texts by leveraging deep neural architectures integrated with attention mechanisms. In this study, we proposed Hybrid deep learning algorithms. CNN, CNN + BiGRU and CNN + BiLSTM with and without an attention-based neural architecture that dynamically focuses on the most important textual features. The proposed hybrid architecture integrates hybrid models with an attention mechanism, allowing the model to capture contextual dependencies which is crucial in legal language understanding. Extensive experiments on a curated dataset of Ethiopian legal texts across multiple classes demonstrate significant improvements on multiple hybrid models like, CNN, CNN + BiGRU and CNN + BiLSTM integrated with Attention mechanism. Model performance is evaluated using an evaluation metrics of precision, recall, F1-score, and accuracy, with evaluation strategies like, 10-fold cross-validation and 80:20 train-test-split which showed notable gains in classification effectiveness. The experimental results show that CNN + BiLSTM with Self-Attention scores 99.53, 99.25, 99.37 and 99.38 for precision, recall, F1-score, and accuracy respectively with 80:20 train-test-split and 99, 98.99, 98.99, and 98.98 for precision, recall, F1-score, and accuracy respectively with 10-fold cross-validation.

## 1. Introduction

In recent years Scholars have shown interest in challenging the historical origins of law and its normative claims to universality [1]*.* By ensuring the impartial and balanced administration of justice and providing remedies for human rights harms, the judiciary contributes significantly to the adjudication and enforcement of human rights. Court reform has been carried out in a number of jurisdictions in an effort to increase access to justice, adjust to changing legal standards, and enhance the court systems overall effectiveness [2]. Many legal traditions address the generality of rules and their exceptions. These issues typically touch on core aspects of the nature and functions of a legal system, such as fairness, coherence, predictability, and fidelity to sources of authority [3–5].

In today’s digital world the rapid digitization of legal documents has created unprecedented opportunities and challenges in the domain of legal informatics, particularly in jurisdictions with low-resource languages like Amharic which stands as the second most widely spoken Semitic language globally, trailing only Arabic [6–8]. Ethiopia’s legal system, comprising a vast and diverse corpus of legal documents from proclamations and regulations to judicial decisions presents a critical need for intelligent systems capable of categorizing texts into relevant legal classes for efficient retrieval, analysis, and decision support. Text classification techniques [9–11], although foundational, often fall short in handling the semantic and syntactic complexity of legal language, especially in morphologically rich and under-resourced languages [7,12–14]. Recent advancements in deep learning have revolutionized natural language processing (NLP), offering powerful models that learn hierarchical and contextual representations from large textual datasets [14–17]. Among these, attention-based architectures have emerged as particularly effective for capturing long-range dependencies and context-sensitive semantics, which are essential for understanding legal discourse. Attention mechanisms, popularized by Transformer-based models, allow systems to selectively focus on relevant parts of the input, making them well-suited for complex document classification tasks where key legal phrases may be dispersed across a lengthy text [8,16,18–21].

Using an attention-based neural architecture, this study presents an improved deep learning method for the multi-class classification of Ethiopian legal texts. To acquire deep contextual features from raw text, our proposed approach uses word embedding and hierarchical attention networks, different than conventional machine learning models that mostly rely on manual feature engineering and shallow representations (TF-IDF). By applying attention layers into a neural architecture, the model enhances interpretability (a critical aspect in legal applications), while preserving word and sentence-level relevance. The article further addresses difficulties with data scarcity and domain adaptation, given the absence of annotated legal datasets in Amharic. We utilize FastText embedding pre-trained on large Amharic corpora to initialize the model and fine-tune it on a curated legal text dataset categorized into multiple classes such as criminal law, civil law, commercial law, Anti-corruption law, Human trafficking law, Labor law, Family law and Constitutional law.

As a whole, this study blends domain-specific data, deep neural architectures, and attention-based learning to offer an appropriate and scalable approach for Ethiopian legal text classification. The findings are likely to have broad impacts on the building of legal research tools, intelligent legal information systems, and AI-assisted decision-support mechanisms that are suitable to the Ethiopian legal system and other undeveloped legal frameworks. Several significant contributions to the field of legal Natural Language Processing (NLP), specifically in low-resource languages, are provided by this study. Following is an outline of our study’s major scientific contributions:

1. A considerable contribution lies in the development and preprocessing of an extensive Ethiopian legal text corpus, tailored for multi-class classification. This involves structured annotation and labeling of legal categories, enabling the training of supervised learning models with a high degree of specificity and contextual alignment.
2. The study introduces attention-based architectures potentially including variants such as CNN, CNN + BiGRU and CNN + BiLSTM with Attention mechanism for contextual relevance in Amharic legal documents. This approach significantly improves the model’s ability to discern semantically salient features critical for multi-class classification tasks.
3. We applied domain-specific embedding from FastText that has already been trained to better represent the linguistic properties of legal terms in Amharic. This indicates how contextualized embedding improved the accuracy of classification, especially for terms with complicated morphology.
4. The framework’s scalability and practical value in real-world lawful data retrieval have been shown by its ability to handle a multi-class classification scenario, where legal texts may fall under numerous and redundant classifications.

The rest of this paper is structured as follows: The Related Work section reviews related work on legal text classification and NLP for Amharic language. The Materials and Methods section outlines the methodology, including data collection, feature extraction, and model selection. The Results and Discussion section presents experimental results and |evaluations. Finally, the conclusion section concludes with key findings and directions for future research.

## 2. Related work

Recent research on text classification spans several thematic directions, particularly in legal text processing, hybrid deep learning architectures, transformer-based models, and domain-specific machine learning approaches. In the legal domain, studies such as [22–25] demonstrate the effectiveness of deep learning for extracting and classifying legal information. For instance, [22] proposed a fuzzy language processing and metaphor recognition framework enhanced with a Bi-LSTM and self-attention mechanism, achieving strong performance in linking legal provisions with court rulings. Similarly, [23] introduced a multi-label legal text classifier that fuses BERT-based embedding with CBOW initialized label vectors to improve multi-label prediction accuracy, while [24] employed a CNN–BiLSTM attention hybrid model and showed that focusing on key legal tokens significantly improved performance and text normalization quality.

Beyond legal applications, hybrid deep learning architectures continue to show superior performance across varied tasks. Studies such as [26] and [27] highlight the advantages of combining CNNs and Bi-LSTMs. In [26] the study reports an accuracy of 91.60% for a CNN–Bi-LSTM sentiment classifier and [27] demonstrating a Deep Parallel Hybrid Fusion Model achieving 90–96% accuracy on disaster-related datasets. Additionally, data augmentation combined with hybrid models, as shown in [28], can substantially enhance robustness and classification accuracy, with WordNet-enriched training data improving macro F1 by up to 20 percentage points when paired with BERT.

A strong emphasis is also placed on transformer-based pre-trained models. In [29], fine-tuned BERT, XLNet, RoBERTa, and ELECTRA models were evaluated for patent classification, with XLNet achieving the highest performance. Likewise, [30] presented the HTMC-PGT framework for hierarchical multi-label classification in poverty governance, integrating XLNet embeddings, Bi-LSTM, hierarchical attention, and transfer learning to achieve a micro-F1 of 96.1%.

Finally, domain-specific machine learning remains influential in contexts such as biomedical and local-language classification. Study [31] showed that TF-IDF representations combined with models like Random Forest and Neural Networks are highly effective for biomedical document classification, particularly for COVID-19-related articles. Similarly, [32] proposed a hybrid MLP–SVM approach for Afaan Oromo health text classification, achieving an accuracy and F1 score of 98.23% and outperforming other classical models.

## 3. Materials and methods

To categorize Amharic textual data, a comprehensive analysis has been carried out by applying algorithms for deep learning such as CNN, CNN + BiGRU and CNN + BiLSTM integrated with Attention mechanism.

### 3.1. Proposed system architecture

**Fig 1:**
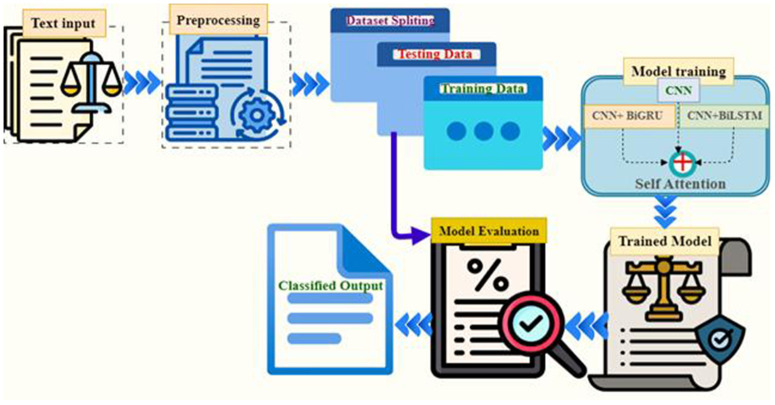
Overall system architecture of the study.

The flow of activities used to develop the proposed Enhanced Automated Classification of Ethiopian Legal Texts is illustrated in Fig 1. The proposed system architecture consists of several steps. The first step involves collecting Ethiopian legal texts written in Amharic script. The next step applies preprocessing activities. The third step involves splitting the labeled datasets into training and testing groups, which are then provided to the selected deep learning classification algorithms to build a model for classifying Ethiopian Legal Texts.

#### 3.1.1. Data collection.

We collected ten (10) Amharic legal documents from the Amhara region Supreme Court, specifically from the south Gondar zone Supreme Court, with eight classes: criminal law, civil law, labor law, family law, constitutional law, Human trafficking law, Anti-corruption law and commercial law. This study aimed to create a model to address the problem of text classification. We created a dataset of 8025 legal sentences extracted from legal documents, manually acquired these documents in Microsoft Word document format from the Supreme Court and associated judges. The dataset comprises eight Classes associated with each sentence, with the sentence itself displaying in the first column and its class being represented by the following columns. We offer a sample of the dataset’s format and structure in Table 1 to help visualization of it.

**Table 1:** Sample Amharic legal sentences showing class labels and corresponding English translations.

Index	Amharic Sentence	English Translation	Class
0	የሙስና ወንጀል በመፈጸሙ በፍትህ አካል እየተፈለገ መሆኑን እያወቀ ወይ	Knowing that he is being sought by the judiciary for committing a corruption crime	Anti-corruption law
1	በመሆኑም አንድ ህገ ወጥ አዘዋዋሪ ወይም ድንበር አሻጋሪ ቁጥራቸው ሁለትና	Therefore, one illegal trafficker or border crosser is two in number	Human trafficking law
2	ወንዱም ሆነ ሴቷ አሥራ ስምንት ዓመት ሳይሞላቸው ጋብቻ መፈፀም አይችሉም	Neither a man nor a woman can marry before they are eighteen years old.	Family law
3	እንደዚሁም አንድ ነጋዴ የንግድ ማኅበር በያንዳንዱ የባጀት ዓመት መጨረሻ	Likewise, a merchant association shall, at the end of each fiscal year	Commercial law
4	በአንድ ክርክር ላይ መፈጸም መደረግ የሚገባው ነገር ሲኖርና ይህም የሚፈጸም	When there is something that needs to be done in a dispute and it is done	Civil law

**Fig 2:**
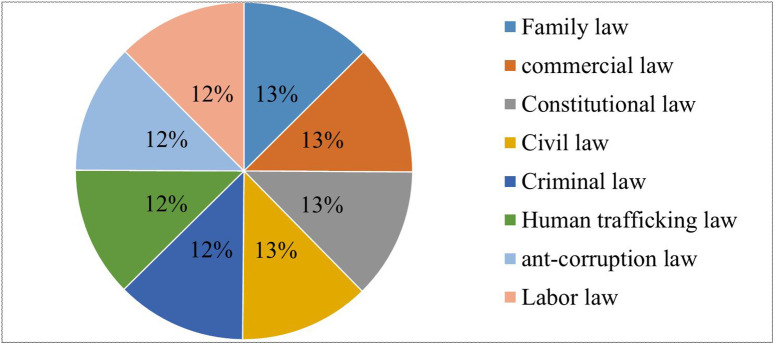
Data Distribution of Ethiopian legal texts.

To construct our dataset, we worked with four annotators for the task. Three of the annotators were Supreme Court employees from the South Gondar zone, and the fourth was a curator who was an expert and previously employee of the Amhara region supreme court, who helped us by reviewing the annotated data and additional case conversations. Without communicating to one another, each annotator annotated the dataset on their own. Lastly, we looked at the majority labels that each annotator contributed to the annotated dataset as shown in Fig 2. The individuals who served as annotators were informed about the purpose of the study, the tasks they were asked to perform, and how their annotation data would be used. All annotators provided informed consent prior to participation. We obtained informed verbal consent from each annotator before they began the annotation task. The research team documented these verbal agreements in a project log, and a member of the team witnessed each consent interaction. No personal, identifiable, or sensitive human data were collected and no minors participated.

#### 3.1.2. Data preprocessing.

Any form of processing performed on raw data to get it ready for next processing step is referred to as preprocessing. Therefore, preparation is the first stage that changes the data into a format that can be processed more quickly and efficiently. The original text must be delivered to NLP in a format that it can use thanks to pre-processing. However, it is a stage in our study that makes our datasets usable by Deep learning algorithms. According to previous studies [32–35] preprocessing plays a critical role in enhancing the performance of deep learning models. In particular, normalization is employed to unify multiple character variants that represent the same phonetic sound into a standard form, while stop-word removal eliminates common and non-informative terms that do not contribute meaningfully to the learning process and stemming is the process of removing infixes, prefixes and suffixes from words to produce the stem or root form.

#### 3.1.3. Model building (training).

**Fig 3:**
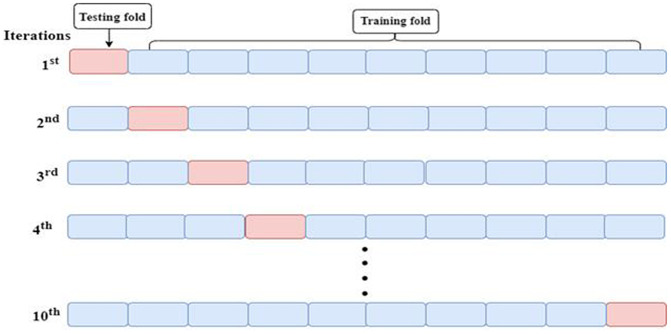
Representation of the dataset in 10 different evaluations.

Numerous train-test splitting techniques were used in the experiment. We agreed on the 80/20 train-test split and 10-fold cross-validation approach. 80:20 train-test split indicates that 20% of the training dataset was employed for model testing and the remaining 80% was used for training. The 80/20 train-test split provided the best outcomes in our experiment. We employed a total of 8025 sentences, of which 6420 were used for training and the remaining 1605 for testing. 10-fold cross-validation is a widely used model evaluation technique designed to provide a more reliable estimate of a model’s generalization performance [32,36]. Cross-validation was employed to obtain a robust estimate of model performance on our relatively small dataset (8,025 sentences). By repeatedly training and evaluating the model on different subsets of the data, it helps reduce variance caused by any particular train-test split and ensures that the reported metrics better reflect the model’s generalization ability. We used 10-fold cross-validation, meaning the dataset was divided into 10 equal parts. In each iteration, 9 folds were used for training and 1-fold for validation, and this process was repeated 10 times so that each fold served as validation once. Fig 3 illustrates that in each evaluation; the system is trained with 90% of dataset samples and validated with the remaining 10%. Therefore, it made training and validation datasets definitely separated from each other.

We built an ensemble of deep learning models that were improved using attention mechanisms in order to attempt an issue of automatically classifying Ethiopian legal writings into multiple classes. For evaluation, three distinct neural network architectures were developed. (i) CNN + Attention Mechanism, (ii) CNN + BiGRU with Attention Mechanism and (iii) CNN + BiLSTM with Attention Mechanism. The attention layer highlighted the most informative tokens in each input sequence, the CNN layer caught n-gram level patterns.

Here, all three models utilize pre-trained FastText embedding to convert each token into a dense vector representation. We downloaded the FastText pretrained embedding model for Amharic language from the link https://fasttext.cc/docs/en/crawl-vectors.html and then used it to generate the vector of words found in our preprocessed data set. FastText embedding are particularly advantageous as they capture sub-word information through character n-grams, allowing the model to generalize better and handle out-of-vocabulary words.

**Fig 4:**
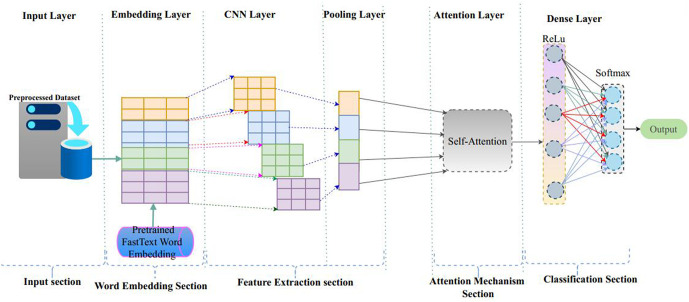
Proposed architecture of CNN + Attention Mechanism.

The provided Fig 4 illustrates a hybrid deep learning model architecture that combines the strengths of Convolutional Neural Networks (CNNs) and a self-attention mechanism for text classification tasks. The model is structured into six distinct sections: the input section, word embedding section, feature extraction section, attention mechanism section, classification section, and output layer, each playing a vital role in transforming raw textual data into accurate predictions. The process begins with the input section, where a preprocessed textual dataset is supplied to the model. The resulting sequence of tokens is then passed to the word embedding section. Following embedding, the feature extraction section applies convolutional neural networks to the embedded input. Multiple convolutional filters are used to detect local patterns such as n-grams within the sequence. Each filter captures different semantic or syntactic features, effectively extracting meaningful local information. This is followed by a pooling layer, typically using max-pooling, which condenses the feature maps by selecting the most prominent features and reducing the dimensionality. The attention mechanism section introduces a self-attention layer that enhances the model’s ability to capture long-range dependencies and contextual relationships between words. Self-Attention is described as a mechanism that computes relationships between all tokens within a sequence, enabling the model to capture global contextual dependencies. In the other hand Hierarchical Attention is defined as a multi-level attention mechanism that operates across different structural levels, assigning importance at each level of the hierarchy.

Unlike convolutional operations that focus on local context, self-attention computes interactions between all tokens in the sequence, enabling the model to assess the relative importance of each word in the broader context of the sentence or document.

Next, in the classification section, the output of the attention mechanism is passed through a series of dense (fully connected) layers. The first dense layer uses a ReLU activation function to introduce non-linearity, which allows the model to learn more complex decision boundaries. These features are then forwarded to a final dense layer equipped with a softmax activation function, which generates a probability distribution over the target classes. This probability distribution forms the basis for the model’s final prediction. The output layer simply selects the class with the highest predicted probability, delivering the final classification result.

**Fig 5:**
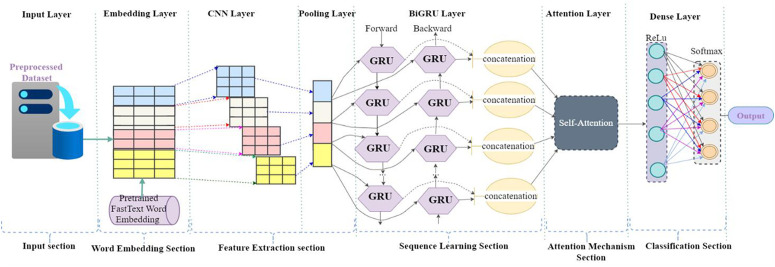
Proposed architeture of CNN + BiGRU with Attention Mechanism.

Fig 5 illustrates a comprehensive hybrid deep learning architecture that integrates Convolutional Neural Networks (CNN), Bidirectional Gated Recurrent Units (BiGRU), and a Self-Attention mechanism to perform text classification. The model is systematically organized into six functional sections: the input section, word embedding section, feature extraction section, classification section and attention mechanism section are the same descriptions of Fig 4 as indicated above and sequence learning section, contributes uniquely to the learning process. The pooled features are forwarded to the sequence learning section, which consists of Bidirectional Gated Recurrent Units (BiGRU). This layer processes the input sequence in both forward and backward directions, enabling the model to capture contextual dependencies from both past and future tokens. The outputs of the forward and backward GRU cells are concatenated to form a rich representation of the input sequence that reflects both directions of temporal information. This dual-context encoding enhances the model’s understanding of language structure and semantics. The concatenated BiGRU outputs are then fed into the attention mechanism section, where a self-attention layer further refines the representation by learning to focus on the most relevant parts of the sequence. Finally, the attention-enhanced features are passed through the classification section, which includes a fully connected dense layer with a ReLU activation function followed by another dense layer with a SoftMax activation function.

**Fig 6:**
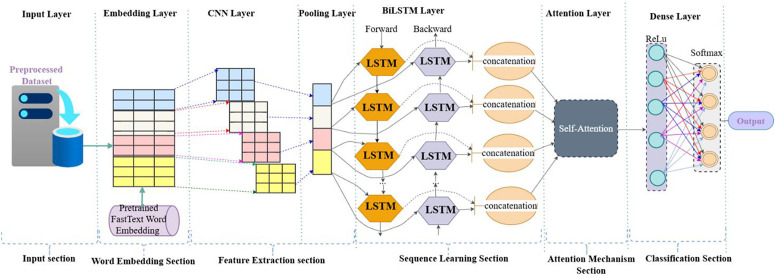
Proposed architeture of CNN + BiLSTM with Attention Mechanism.

The given architecture diagram represents as indicated in Fig 6 proposed a hybrid deep learning model that combines Convolutional Neural Networks (CNN), Bidirectional Long Short-Term Memory (BiLSTM) networks, and a Self-Attention mechanism for text classification. This model is structured into several logical sections: the input section, word embedding section, feature extraction section, classification section and attention mechanism section are the same descriptions of Fig 4 as indicated above and sequence learning section, contributes uniquely to the learning. The unique section of the proposed models was on sequence learning section, which leverages Bidirectional LSTM (BiLSTM) networks to learn long-range dependencies in the text. Unlike unidirectional LSTMs, BiLSTM layers process sequences in both forward and backward directions. This bidirectional context enables the model to capture comprehensive sequential information, including dependencies from both past and future tokens. The forward and backward LSTM outputs are then concatenated, yielding a rich contextual representation for each word in the input sequence. Subsequently, the attention mechanism section applies a self-attention layer to the BiLSTM outputs.

From this end, all models included a max-pooling or attention-weighted pooling layer, followed by one or more fully connected dense layers and a final softmax activation layer to handle multi-class predictions. The attention mechanism was implemented as a trainable layer that calculated the importance of each hidden state and produced a context vector, which acted as a weighted sum of the sequence outputs, thereby improving the model’s interpretability and focus on key textual components.

#### 3.1.4. Experimental setup.

To carry out the tasks at hand, from data loading to model evaluation, we made use of a generous 12 GB RAM and GPU from the Google collaborator. Initially, we annotated (labeled) the data in a CSV file. Following that, we uploaded data to Google Drive in CSV file format. The uploaded CSV file was then utilized by mounting the Google disk to Google Collab. The necessary Tensor Flow and Keras libraries are imported after mounting the Google drive. Loading CSV into the Collaborator environment was the next step. The loaded CSV file needs to be divided into training and testing datasets of 80% and 20%, respectively. The most basic measures used to measure model performance evaluation are F1-Score, precision, recall and accuracy. Table 2 presents a summary of the hyper-parameters chosen for this study using trial and error or manual method. The parameters significantly influence model accuracy. This approach was implemented to accelerate training, as utilizing the complete dataset would have necessitated considerably more time. This smaller subset may result in over-fitting, thereby diminishing generalization to new data.

**Table 2:** Parameters and their respective values for this study.

Hyper-parameters	Value
Embedding dimension	300
Kernel size	5
Drop out	0.5
Optimizer	Adam
Activation	ReLu
Epochs	12
Batch size	32
Max_vocab	10000

**3.1.4.1. Model Evaluation metrics.** There are two types of classification problems depending on the number of classes: binary classification, where there are only two classes, and multi-class classification, where there are more than two classes [37,38]. This study used multi-class classification to evaluate the proposed models with eight class Ethiopian legal texts and we used evaluation metrics to evaluate the performance which include accuracy, precision, recall, and F1 score given by equations (1)–(4).


$$Accuracy=\frac{TP+TN}{TP+TN+FPFN}$$ (1)

$$Precision=\frac{TP}{TP+FP}$$ (2)

$$Recall=\frac{TP}{TP+FN}$$ (3)

$$F{\text{1}}_{Score}=\frac{\text{2}*Precision*Recall}{Precision+Recall}$$ (4)

## 4. Results and discussion

This section presents the implemented models and the experiments with the result obtained for evaluating the performance of the study. Regarding the problems we studied and comparing convolutional recurrent neural network (CNN) integrate with Bidirectional Gated Recurrent Units (CNN + BiGRU), CNN combined with Bidirectional Long Short-Term Memory (CNN + BiLSTM) and the Attention module. To do so, we need to have analysis of our dataset that we used for implementing our model. In this study, two major experimental evaluation strategies were employed to assess the performance and generalization ability of the proposed models: 10-fold cross-validation and an 80:20 train–test split. The 10-fold cross-validation approach involved dividing the dataset into ten equal subsets and iteratively training the model on nine folds while using the remaining fold for testing. In contrast, the 80:20 train–test split provided a common evaluation setting, where 80% of the data was used for training and the remaining 20% served as an independent test set.

Table 3 indicates the cross-validation results which demonstrate a consistent improvement in performance as additional sequence-modeling layers are incorporated. Under 10-fold cross-validation, the baseline CNN model achieves strong and stable values, with a precision of 97.37, recall of 97.32, F1-score of 97.33, and accuracy of 97.32. Introducing a BiGRU layer further boosts performance, with the CNN + BiGRU model reaching a precision of 97.90 and an F1-score and accuracy of 0.9785. The highest cross-validation scores are achieved by the CNN + BiLSTM model, which attains a precision of 99.00, recall of 98.99, F1-score of 98.99, and accuracy of 98. 98. Overall, the cross-validation evaluation confirms that hybrid architectures, especially CNN + BiLSTM, provide superior generalization and robustness compared to a standalone CNN.

**Table 3:** Comparison of model performance using 10-fold cross-validation with attention mechanism.

Models	Evaluation Metrics			
	Precision	Recall	F1-score	Accuracy
CNN	97.37	97.32	97.33	97.32
CNN + BiGRU	97.90	97.85	97.85	97.85
CNN + BiLSTM	99	98.99	98.99	98.98

Table 4 presents the results for three models: a baseline Convolutional Neural Network (CNN), CNN integrate with Bidirectional Gated Recurrent Units (CNN + BiGRU), and CNN combined with Bidirectional Long Short-Term Memory (CNN + BiLSTM). The baseline CNN model achieved strong performance, with a precision of 96.45%, recall of 96.49%, F1-score of 96.25%, and accuracy of 96.51%. This indicates that the CNN is capable of effectively capturing spatial features from the input text sequences. However, CNN alone may struggle to capture long-range dependencies or contextual information due to its limited sequential modeling capability.

**Table 4:** Model performance comparisons using hold-out (80:20) without Attention Mechanism.

Models	Evaluation Metrics			
	Precision	Recall	F1-score	Accuracy
CNN	96.45	96.49	96.25	96.51
CNN + BiGRU	96.58	96.57	96.60	96.69
CNN + BiLSTM	96.71	96.62	96.72	96.76

To address these issues, we integrated bidirectional recurrent layers with CNN. The CNN + BiGRU model showed slight improvements across all metrics: precision (96.58%), recall (96.57%), F1-score (96.60%), and accuracy (96.69%). The CNN + BiLSTM model further improved performance, achieving the highest scores in all evaluation metrics: precision (96.71%), recall (96.62%), F1-score

(96.72%), and accuracy (96.76%). This suggests that BiLSTM layers are more effective in capturing complex contextual relationships in Amharic texts. When combined with CNN, the model benefits from both spatial and temporal feature extraction, leading to a more robust and accurate classification. Overall, the results demonstrate that incorporating recurrent layers, especially BiLSTM, enhances the performance of CNN-based models for Amharic text classification. Among the evaluated architectures, CNN + BiLSTM consistently outperforms the others, making it the most suitable model for this task.

Table 5 presents the performance of three attention-mechanism models: CNN, CNN + BiGRU, and CNN + BiLSTM, evaluated using standard metrics Precision, Recall, F1-score, and Accuracy. The CNN model with attention achieved a precision of 97.73%, recall of 97.49%, F1-score of 97.62%, and an accuracy of 97.69%. These scores show a marked improvement over its non-attention counterpart, confirming that the attention mechanism contributes to more effective feature prioritization, even in the absence of recurrent structures. The CNN + BiGRU with attention model outperformed the baseline CNN by a significant margin, with precision of 98.12%, recall of 98.24%, F1-score of 98.12%, and accuracy of 98.25%. The integration of BiGRU enables the model to learn sequential dependencies in both directions, while attention refines the importance of each token within its context.

**Table 5:** Model performance comparisons hold-out (80:20) with Attention Mechanism.

Models	Evaluation Metrics			
	Precision	Recall	F1-score	Accuracy
CNN+ Attention	97.73	97.49	97.62	97.69
CNN + BiGRU+ Attention	98.12	98.24	98.12	98.25
CNN + BiLSTM+ Attention	99.53	99.25	99.37	99.38

Notably, the CNN + BiLSTM with attention model achieved the best overall results, with precision of 99.53%, recall of 99.25%, F1-score of 99.37%, and accuracy of 99.38%. The exceptional performance of this model can be attributed to the complementary strengths of BiLSTM’s long-range dependency modeling and the attention mechanism’s selective focus. This combination allows the model to extract fine-grained semantic and syntactic patterns critical for classifying complex linguistic structures in Amharic legal or formal texts. In summary, the CNN + BiLSTM with attention configuration standout as the most effective model, making it a compelling choice for high-accuracy Amharic text classification tasks.

**Fig 7:**
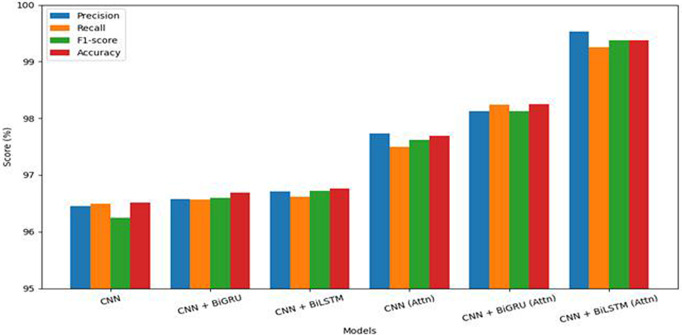
Model performance comparisons with and without Attention Mechanism.

Fig 7 presents a comparative analysis of six deep learning architectures evaluated using four key performance metrics: Precision, Recall, F1-score, and Accuracy. These models are divided into two categories: those without attention mechanisms (CNN, CNN + BiGRU, and CNN + BiLSTM) and those combined with attention mechanisms (CNN (Attention), CNN + BiGRU (Attention), and CNN + BiLSTM (Attention)). Among the models without attention, the CNN + BiLSTM variant achieves the highest performance across all metrics, indicating that adding a bidirectional LSTM layer improves the model’s ability to capture contextual dependencies in the input data. However, the baseline CNN model performs the lowest in all metrics, highlighting the limitations of using a purely convolutional approach for sequential data tasks such as text classification.

Attention mechanisms lead to a marked improvement across all model variants. Notably, the CNN + BiLSTM with attention outperforms all other models, achieving a precision of 99.53%, recall of 99.25%, F1-score of 99.37%, and accuracy of 99.38%. This substantial improvement indicates the effectiveness of combining bidirectional LSTM layers with attention mechanisms, allowing the model to better focus on informative words or phrases in a sequence. Similarly, the CNN + BiGRU with attention also shows strong performance, exceeding all models without attention and closely approaching the performance of the BiLSTM-based attention model. Overall, the results demonstrate that the inclusion of attention mechanisms consistently enhances model performance, with improvements observed across all evaluation metrics. The results clearly indicate that the integration of attention mechanisms, particularly in combination with BiLSTM layers, is a highly effective strategy for improving classification performance in deep learning models applied to sequential text data.

**Fig 8:**
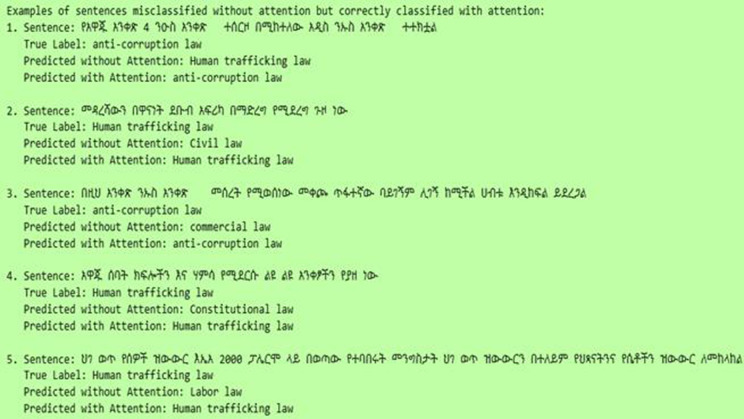
Wrongly and correctly predicted labels with and without Attention Mechanisms.

Fig 8 presents representative examples of Amharic legal sentences that were misclassified by the baseline model without attention but correctly classified after incorporating the attention mechanism. For each instance, the true label, the prediction without attention, and the prediction with attention are provided for comparison. The results describe that the model without attention tends to confuse semantically related legal categories, such as human trafficking law, civil law, and constitutional law. However, with the integration of the attention mechanism, the model is able to correctly identify the relevant class across all presented cases. This improvement suggests that attention enables the model to focus on more informative and contextually relevant terms within the sentence.

In particular, the corrected predictions indicate that attention helps disambiguate complex legal expressions by assigning higher importance to discriminative keywords associated with specific legal domains (terms related to corruption or human trafficking). This behavior supports the claim that the attention mechanism enhances the model’s ability to capture contextual dependencies in Amharic legal text. All in all, these examples provide qualitative evidence of the effectiveness of attention in improving classification performance, especially in challenging cases where different legal categories exhibit overlapping vocabulary.

The experimental results show in Table 6 incorporating an attention module consistently improves the performance of all models across both macro and weighted F1-scores. The baseline CNN model increases from 96.78 to 98.43 (macro F1) and from 97.31 to 98.00 (weighted F1) when attention is added. The CNN + BiGRU model also benefits, improving from 97.65 to 98.57 (macro F1) and maintaining a strong weighted F1 of 98.00 with attention. The largest gain is observed in the CNN + BiLSTM model, where the macro F1-score rises from 97.74 to 99.32 and the weighted F1-score from 97.72 to 99.37. Overall, the attention mechanism enhances each architecture, with the CNN + BiLSTM model achieving the highest performance.

**Table 6:** Comparison of Models with Macro and Weighted average of F1-Score.

Models	With out Attention Module		With Attention Module	
	Macro Average F1-Score	Weighted Average F1-Score	Macro Average F1-Score	weighted Average F1-Score
**CNN**	96.78	97.31	98.43	98
**CNN + BiGRU**	97.65	97.55	98.57	98
**CNN + BiLSTM**	97.74	97.72	99.32	99.37

**Fig 9:**
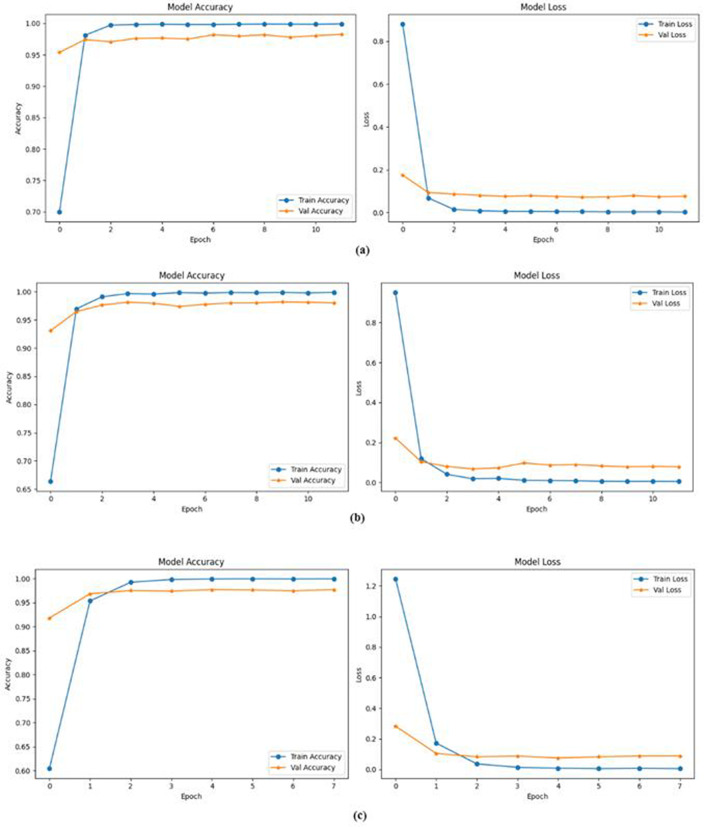
Accuracy and Loss versus epoch plot: (a) for CNN + BiGRU + Self-Attention, (b) for CNN + BiLSTM + Self-Attention and (c) for CNN + Self-Attention.

As illustrated in Fig 9, all models demonstrated rapid convergence within the first few epochs, with training accuracy reaching near-perfect levels (above 99%) by epoch 2 or 3. This indicates that each architecture was highly effective in fitting the training data. Correspondingly, the training loss dropped sharply to near-zero levels in the early stages of training, confirming the models’ learning efficiency. Among the models, the CNN + BiGRU + Self-Attention architecture as indicated in Fig 9 subfigure (a) achieved the highest validation accuracy, approximately 98%, and exhibited the most stable loss curve, suggesting strong generalization and robustness. The inclusion of BiGRU allowed the model to capture complex temporal dependencies in the data, while the self-attention mechanism enhanced feature representation by dynamically focusing on the most relevant input regions. The CNN + BiLSTM + Self-Attention model as indicated in Fig 9 subfigure (b) also performed well, with validation accuracy exceeding 97%, although it displayed slightly more variation in validation loss. This minor fluctuation may be attributed to the longer memory nature of LSTM units, which can lead to increased sensitivity in training.

Interestingly, the CNN + Self-Attention model as indicated in Fig 9 subfigure (c), despite lacking a recurrent component, maintained competitive performance with validation accuracy around 97%. This result is significant as it demonstrates that self-attention mechanisms alone, when combined with CNN feature extraction, can yield high classification performance while reducing computational complexity. Across all models, the validation loss stabilized after the initial epochs, indicating convergence and consistent performance. However, the small but persistent gap between training and validation accuracy points to a degree of overfitting, which may be mitigated in future work through the use of dropout, L2 regularization, or more extensive data augmentation.

Overall, these results highlight the effectiveness of combining CNNs with self-attention and recurrent layers. The self-attention mechanism proves particularly valuable in enhancing the model’s focus on informative features, while recurrent units contribute to capturing sequence-level dependencies. The comparative performance suggests that while BiGRU slightly outperforms BiLSTM in terms of stability and generalization, even a non-recurrent model with self-attention can achieve competitive results, making it an attractive option when computational efficiency is a concern.

The results reported in Tables 7–9 demonstrate consistently high performance across all evaluated models, with overall accuracy reaching up to 99% and uniformly strong precision, recall, and F1-scores across all legal categories. While these results are encouraging, such near-perfect performance raises concerns regarding potential overfitting, particularly given the relatively limited size of the dataset. Furthermore, the performance differences among the models are marginal, with only slight improvements observed when transitioning from CNN+Self-Attention to more complex architectures such as CNN + BiGRU+Self-Attention and CNN + BiLSTM+Self-Attention. This makes it difficult to clearly justify the added architectural complexity in terms of meaningful performance gains. In particular, the improvements in F1-score are minimal and may not be statistically significant, suggesting that the increased model complexity may not translate into substantial practical benefits.

**Table 7:** Classification Report for CNN+ Self Attention.

Class	precision	recall	f1-score	support
Civil law	0.96	0.96	0.96	201
Constitutional law	0.97	0.97	0.97	201
Criminal law	0.99	0.98	0.99	200
Family law	0.97	0.99	0.98	202
Human trafficking law	0.99	0.97	0.98	200
Labor law	0.99	0.98	0.99	200
ant-corruption law	0.99	1.00	0.99	200
commercial law	0.96	0.95	0.95	201
accuracy			0.98	1605
macro avg	0.98	0.98	0.98	1605
weighted avg	0.98	0.98	0.98	1605

**Table 8:** Classification Report for CNN + BiGRU+Self Attention.

Class	precision	recall	f1-score	support
Civil law	0.96	0.99	0.97	201
Constitutional law	0.99	0.99	0.99	201
Criminal law	0.98	0.98	0.98	200
Family law	0.99	0.98	0.98	202
Human trafficking law	0.99	0.99	0.99	200
Labor law	0.99	0.99	0.99	200
ant-corruption law	0.98	0.97	0.98	200
commercial law	0.97	0.97	0.97	201
accuracy			0.98	1605
macro avg	0.98	0.98	0.98	1605
weighted avg	0.98	0.98	0.98	1605

**Table 9:** Classification Report for CNN + BiLSTM+Self Attention.

Class	precision	recall	f1-score	support
Civil law	0.98	1.00	0.99	182
Constitutional law	0.98	0.99	0.99	199
Criminal law	1.00	0.98	0.99	200
Family law	1.00	0.99	0.99	207
Human trafficking law	1.00	1.00	1.00	216
Labor law	1.00	0.99	1.00	194
ant-corruption law	1.00	0.99	0.99	203
commercial law	1.00	1.00	1.00	204
accuracy			0.99	1605
macro avg	0.99	0.99	0.99	1605
weighted avg	0.99	0.99	0.99	1605

**Fig 10:**
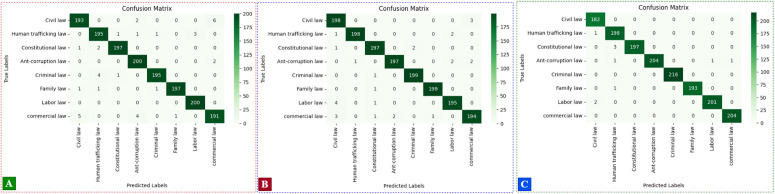
Confusion Matrices of Attention Mechanism with three hybrid deep learning algorithms: (A) CNN + Self-Attention, (B) CNN  +  BiGRU  +  Self-Attention, (C) CNN  +  BiLSTM  +  Self-Attention.

Further analysis was conducted using confusion matrices to evaluate the class-wise performance of each model, as shown in Fig 10. The confusion matrices confirm that all three hybrid models consistently achieve high accuracy across the eight legal categories. Notably, the CNN + Self-Attention model as indicated in Fig 10 subfigure (A), achieves the most balanced distribution with fewer off-diagonal errors overall. It excels particularly in the classification of Anti-Corruption Law and Criminal Law, where near-perfect predictions are observed. This result indicates that even without recurrent units, the self-attention mechanism is capable of capturing nuanced relationships in the data, resulting in highly accurate predictions.

The CNN + BiGRU + Self-Attention model as indicated in Fig 10 subfigure (B), demonstrates strong class-wise performance, though minor misclassifications are observed, particularly between Commercial Law and Civil Law, where semantic overlap may cause confusion. Similarly, the CNN + BiLSTM + Self-Attention model as indicated in Fig 10 subfigure (C), yields slightly improved performance in distinguishing Human Trafficking Law and Criminal Law, suggesting that the BiLSTM layer effectively captures contextual dependencies specific to these categories. Across all models, the most frequently misclassified categories tend to be those with thematic or terminological similarities, such as Civil Law and Commercial Law, or Family Law and Labor Law. This demonstrates the robustness of the proposed architectures and confirms their suitability for legal document classification tasks. The confusion matrix analysis complements the accuracy and loss evaluations.

**Fig 11:**
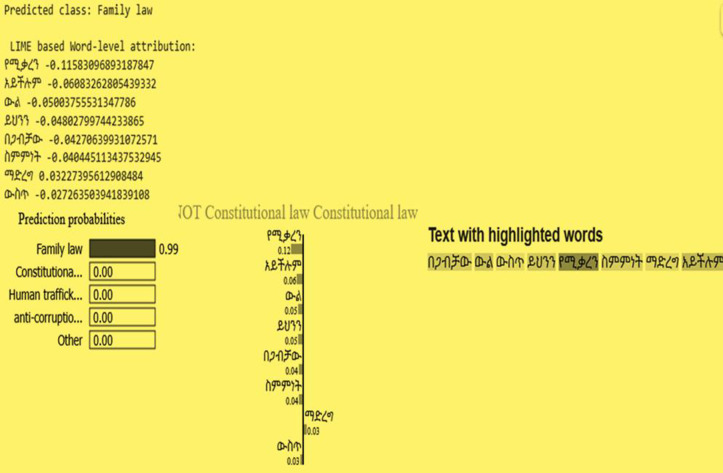
Graphical representation of model interpretability using LIME.

Fig 11 illustrates a representative example of word-level interpretability generated using LIME (Local Interpretable Model-Agnostic Explanations) for an Amharic legal text instance. The model predicts the input sentence as belonging to the Family law category, as indicated at the top of the figure. The accompanying visualization highlights the contribution of individual words to the model’s decision, where each token is assigned an importance score reflecting its influence on the predicted class.

This interpretability analysis provides empirical evidence that the model extracted meaningful lexical cues within Amharic legal syntax. However, it is also observed that only a limited number of tokens dominate the decision-making process, which may suggest a reliance on key terms rather than broader contextual understanding. Therefore, while the results support the effectiveness of the model in identifying discriminative features, further analysis such as combining LIME with attention weight visualization could provide deeper insights into contextual dependency modeling. Table 10 shows how our study’s model comparison utilizing accuracy is depicted.

**Table 10:** Comparison of the proposed model with existing state-of-art models.

Article References	Tasks	Model	Model Accuracy	
			Without Attention	With Attention
[26]	Sentiment Analysis	CNN-BiLSTM	91.60	---
[33]	Categorization	BERT	88%	---
[34]	Sentiment Analysis	CNN-BiLSTM	96.08%	---
[39]	Classification	CNN-BiLSTM	89.06	91.41
[40]	Classification	BERT	93.77	---
[41]	Categorization	CNN	93.79%	---
[42]	Classification	BERT	91.77	---
[43]	Sentiment Analysis	Hybrid	97.86	---
[44]	Classification	Hybrid	97.69	96.37
[45]	Classification	BiLSTM	88.00	85.00
**Proposed study**	**Classification**	**CNN** **CNN + BiGRU** **CNN + BiLSTM**	**96.51** **96.69** **96.76**	**97.69** **98.25** **99.38**

## 5. Conclusion

This study has introduced a robust and effective deep learning framework tailored for the nuanced task of multi-class classification of Ethiopian legal texts. By leveraging hybrid neural architectures namely CNN, CNN + BiGRU, and CNN + BiLSTM with and without attention mechanisms, the proposed models effectively address the complex semantics and structural intricacies inherent in legal language. The integration of attention mechanisms, particularly self-attention, enhances the model’s ability to focus on contextually salient features, thereby improving the interpretability and performance of the classification process. Model performance was evaluated using an evaluation metrics of precision, recall, F1-score, and accuracy, with evaluation strategies like, 10-fold cross-validation and 80:20 train-test-split which showed notable gains in classification effectiveness. Experimental results demonstrate that the CNN + BiLSTM model augmented with self-attention achieves exceptional performance, attaining precision, recall, F1-score, and accuracy all above 99% for both evaluation strategy, underscoring its potential for practical deployment in real-world legal document analysis systems. Overall, this research contributes a scalable and high-performing approach to legal text classification in underrepresented languages and domains, setting a foundation for future advancements in legal AI applications within the Ethiopian judicial context and beyond.
